# Supplementary material for: A series of dual‐reporter vectors for ratiometric analysis of protein abundance in plants
Source: Plant Direct. 2020 Jun 21;4(6):e00231. doi: 10.1002/pld3.231 (PMC7306620; doi:10.1002/pld3.231)
Supplement: Supplementary file 1 — Fig S1‐S7 [file PLD3-4-e00231-s001.pdf]

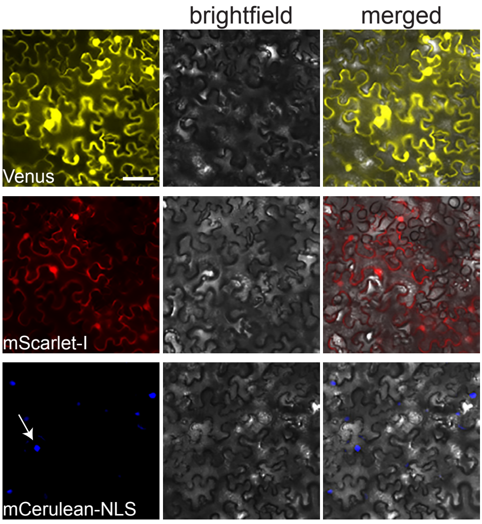

Supplemental Figure 1. Fluorescence microscopy images showing the expression of reference proteins in *N. benthamiana* epidermal cells.

Epifluorescence microscopy images of *N. benthamiana* epidermal cells transformed with pRATIO2212, 2231, and 2214, expressing Venus (yellow), mScarlet-I (red), and mCeruelan-NLS (blue) as reference proteins, respectively. Arrow indicates nuclear localization. Scale bar = 55 µm.

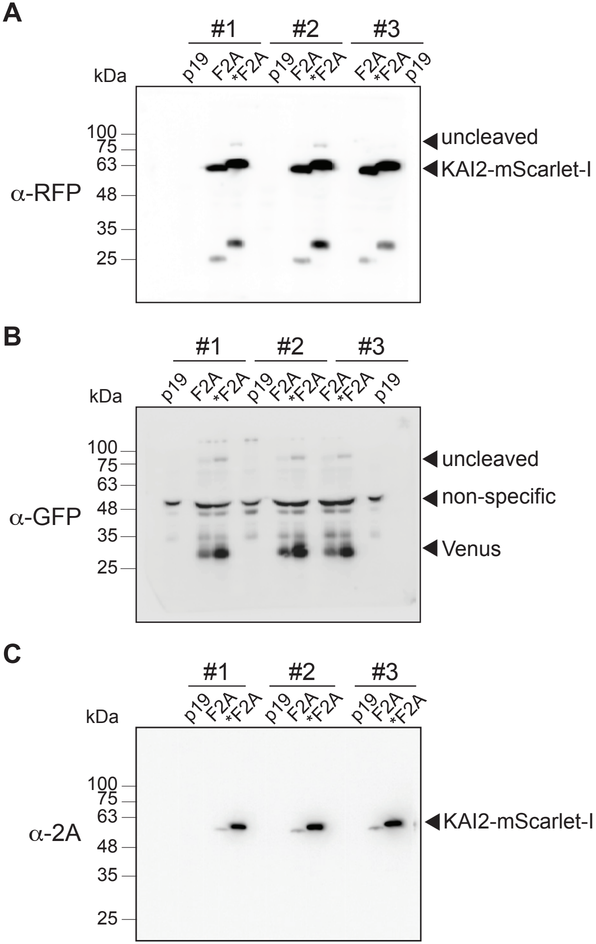

Supplemental Figure 2. Western blot analysis revealing cleavage efficiency in the 2As in *N. benthamiana* epidermal cells.

Proteins obtained from three independent biological replicates of each F2A and *F2A samples were probed against **(A)** mScarlet-I, **(B)** Venus, and **(C)** 2A peptide. The cleavage efficiency was assessed as shown in Figure 2D. Leaf transformed with p19 served as the negative control.

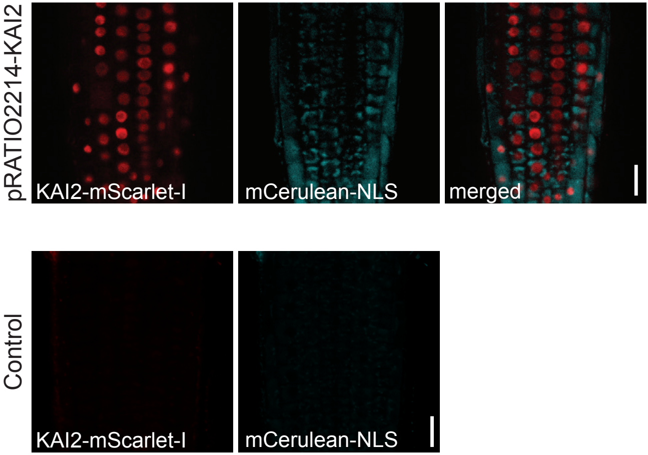

Supplemental Figure 3. Reference protein is undetectable in *Arabidopsis thaliana*.

Representative images of Arabidopsis root meristem in T2 seedlings expressing KAI2-mScarlet-I fusion protein from the *UBQ10* promoter. The control represents a non-fluorescent seedling from the segregating T2 population. Scale bar: 50 µm

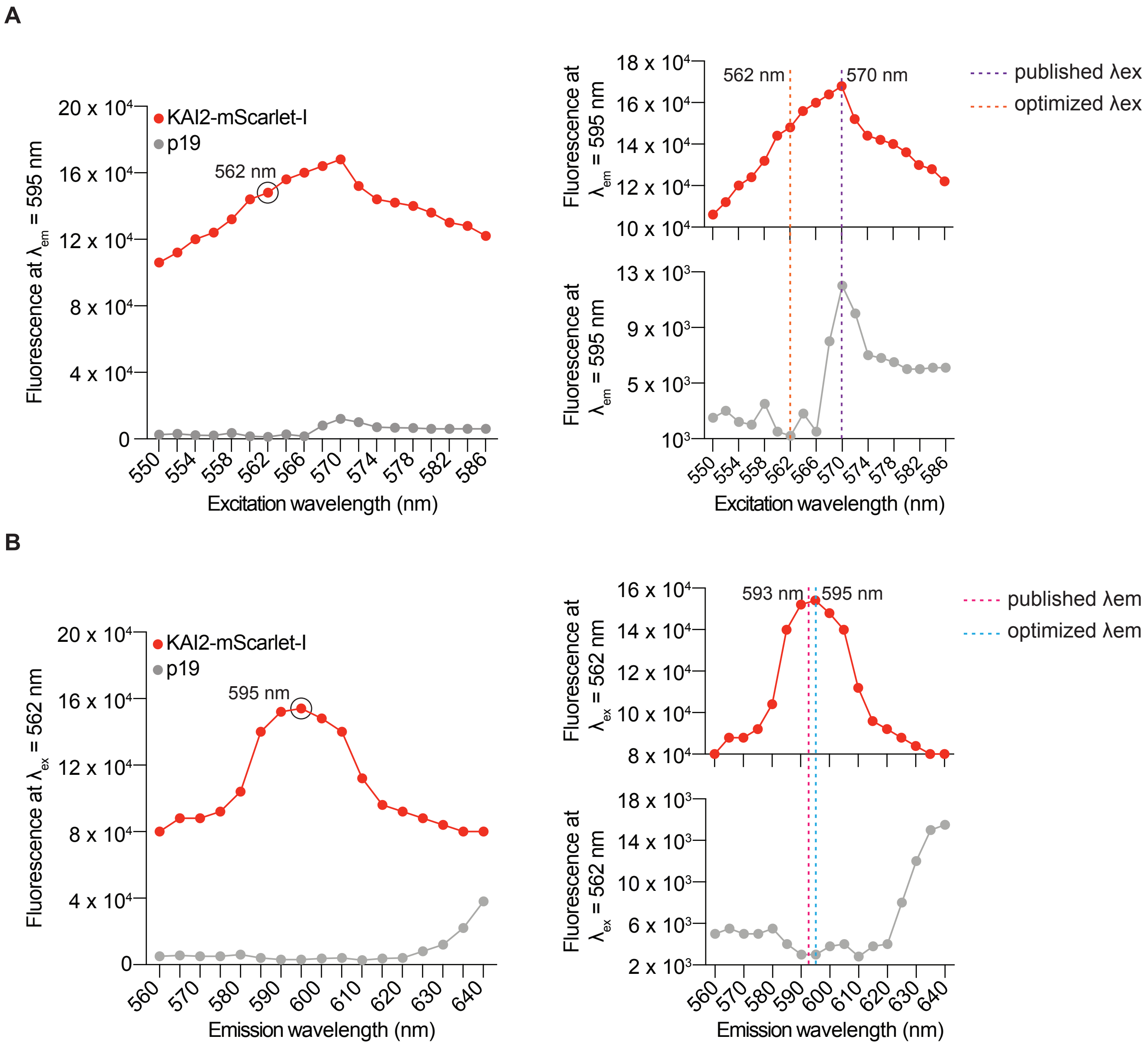

Supplemental Figure 4. Excitation and emission spectra of mScarlet-I in *N. benthamiana*.
Excitation **(A)** and emission **(B)** spectra of mScarlet-I were recorded with a multi-mode plate reader (CLARIOstar) (excitation/emisssion slit: 1.0 nm). The left panel shows the spectra for the sample KAI2-mScarlet-I (red) and background p19 spectrum (grey) plotted on the same graph, while the right panel shows the same data plotted on separate graphs.

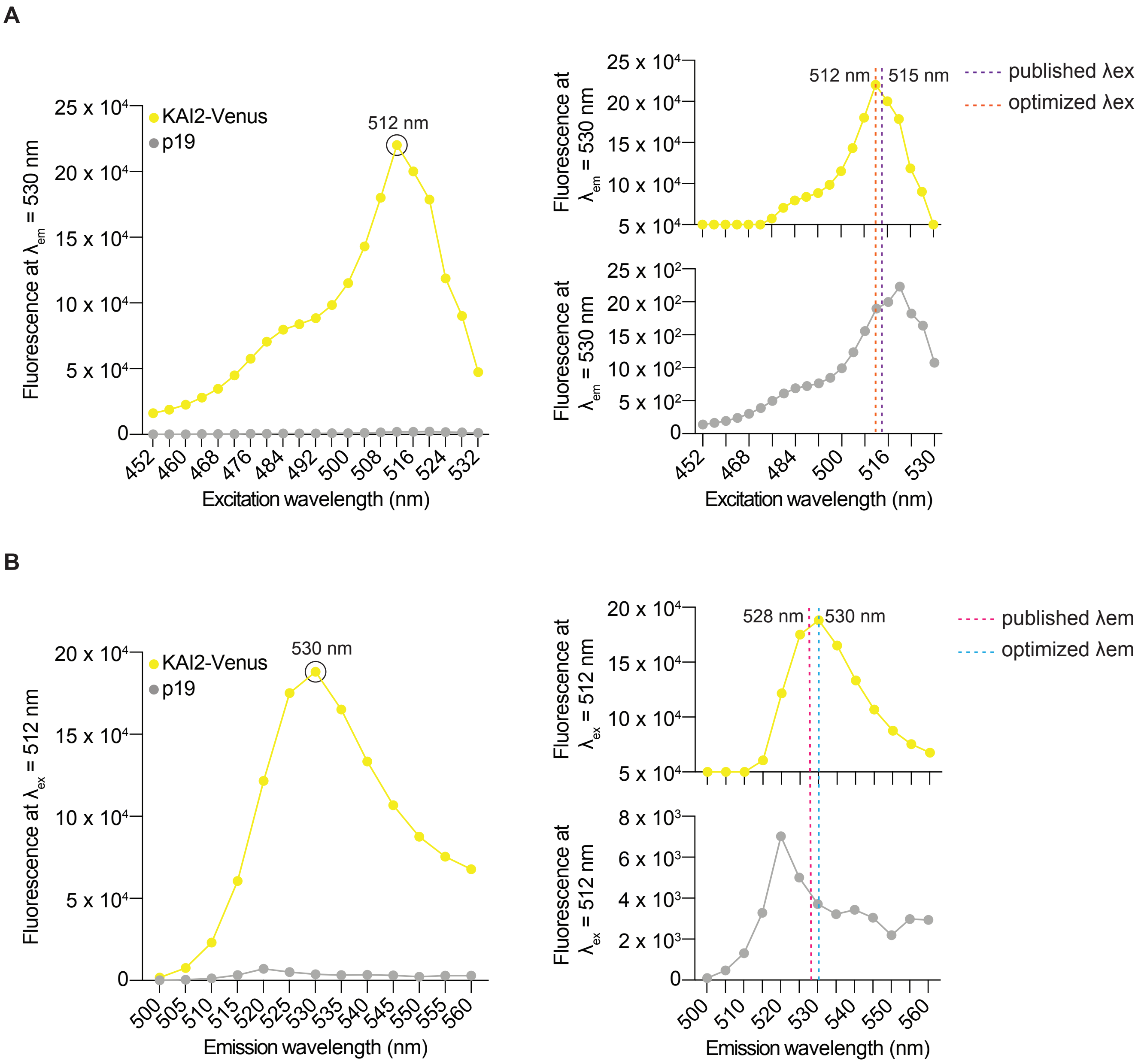

Supplemental Figure 5. Excitation and emission spectra of Venus in *N. benthamiana*.
Excitation **(A)** and emission **(B)** spectra of Venus. Presentation of data is the same as for Supplemental Figure 4, but with KAI2-Venus (yellow).

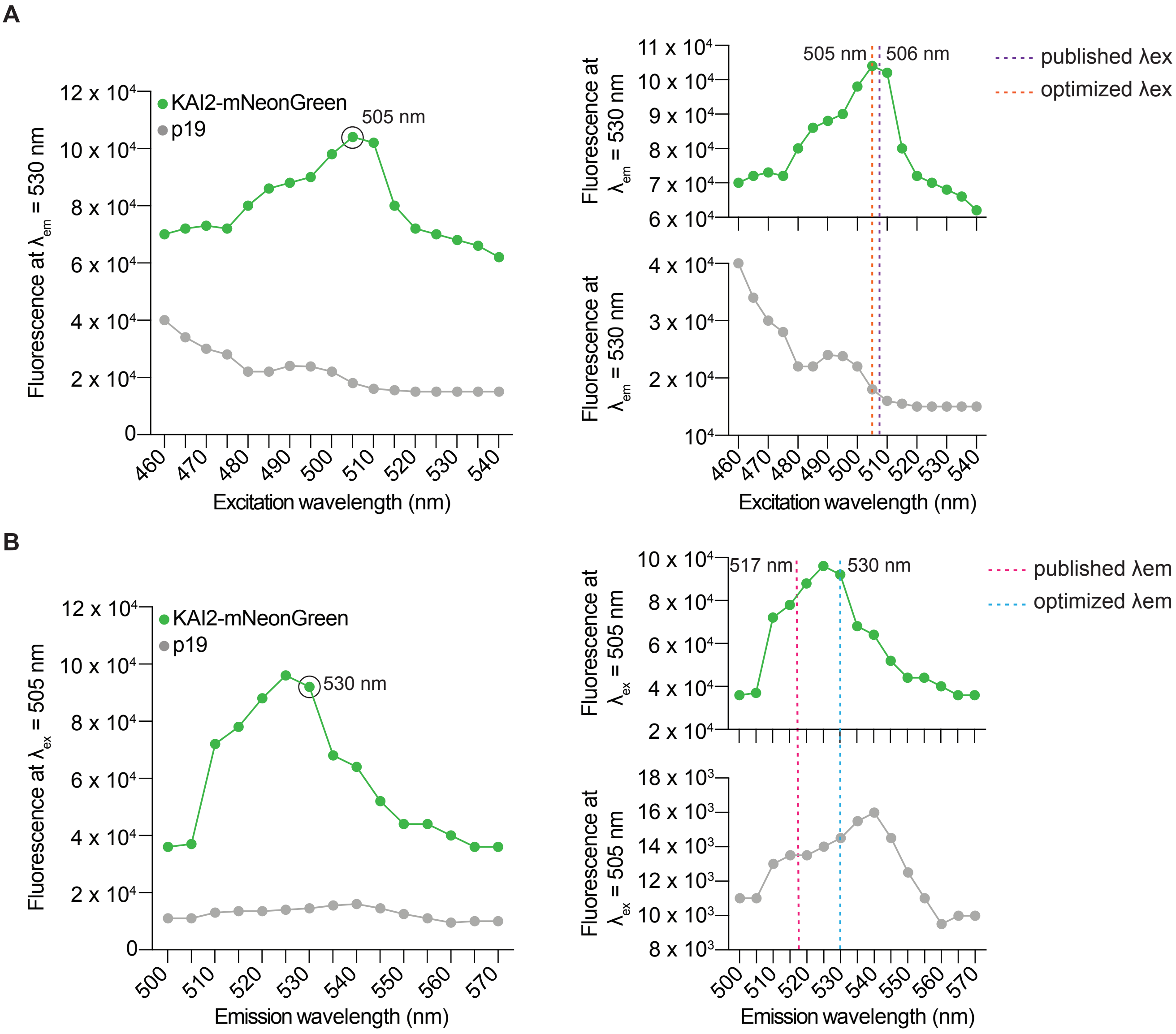

Supplemental Figure 6. Excitation and emission spectra of mNeonGreen in *N. benthamiana*.
Excitation **(A)** and emission **(B)** spectra of mNeonGreen. Presentation of data is the same as for Supplemental Figure 4, but with KAI2-mNeonGreen (green).

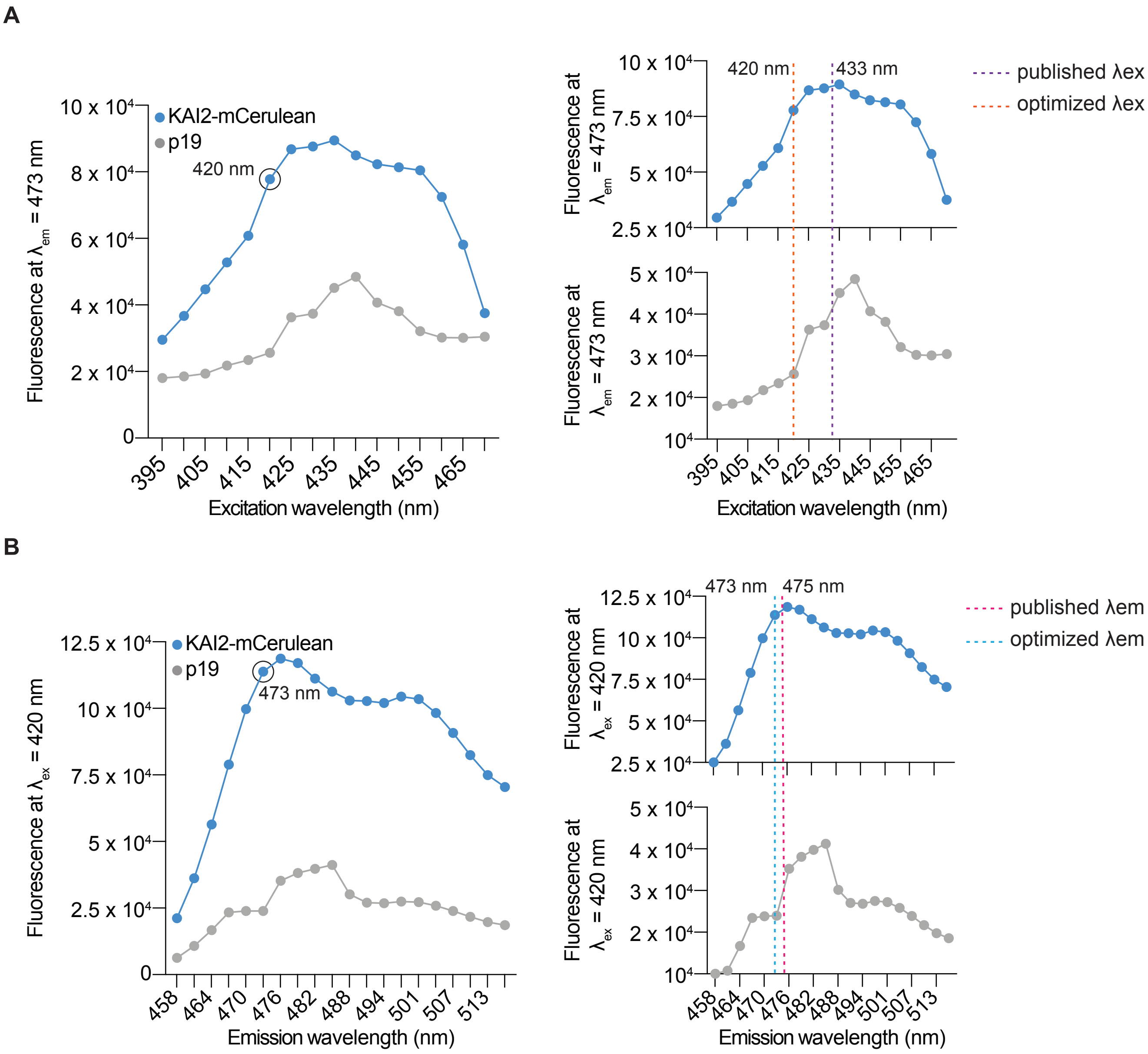

Supplemental Figure 7. Excitation and emission spectra of mCerulean in *N. benthamiana*.
Excitation **(A)** and emission **(B)** spectra of mCerulean. Presentation of data is the same as for Supplemental Figure 4, but with KAI2-mCerulean (blue).
