# Supplementary material for: A series of dual‐reporter vectors for ratiometric analysis of protein abundance in plants
Source: Plant Direct. 2020 Jun 21;4(6):e00231. doi: 10.1002/pld3.231 (PMC7306620; doi:10.1002/pld3.231)
Supplement: Supplementary file 2 — Table S1 [file PLD3-4-e00231-s002.pdf]

Table S1. List of vectors in the pRATIO series.

pRATIO	Promoter	NLS (+/-)	Target	2A	Reference	Selection marker (Bacteria/Plants)
**pRATIO1112**	*35Sp*	+	mScarlet-I	F2A	Venus	Spec/Kan
**pRATIO1131**	*35Sp*	+	mNeonGFP	F2A	mScarlet-I	Spec/Kan
**pRATIO1151**	*35Sp*	+	LUC2	F2A	mScarlet-I	Spec/Kan
**pRATIO2112**	*UBQ10p*	+	mScarlet-I	F2A	Venus	Spec/Kan
**pRATIO2131**	*UBQ10p*	+	mNeonGFP	F2A	mScarlet-I	Spec/Kan
**pRATIO2151**	*UBQ10p*	+	LUC2	F2A	mScarlet-I	Spec/Kan
**pRATIO1212**	*35Sp*	+	mScarlet-I	*F2A	Venus	Spec/Kan
**pRATIO1251**	*35Sp*	+	LUC2	*F2A	mScarlet-I	Spec/Kan
**pRATIO1267**	*35Sp*	+	redLUC	*F2A	gLUC	Spec/Kan
**pRATIO2212**	*UBQ10p*	+	mScarlet-I	*F2A	Venus	Spec/Kan
**pRATIO2214**	*UBQ10p*	+	mScarlet-I	*F2A	mCerulean	Spec/Kan
**pRATIO2231**	*UBQ10p*	+	mNeonGFP	*F2A	mScarlet-I	Spec/Kan
**pRATIO2251**	*UBQ10p*	+	LUC2	*F2A	mScarlet-I	Spec/Kan
**pRATIO3212**	*35Sp*	-	mScarlet-I	*F2A	Venus	Spec/Kan
**pRATIO3267**	*35Sp*	-	redLUC	*F2A	gLUC	Spec/Kan
**pRATIO4212**	*UBQ10p*	-	mScarlet-I	*F2A	Venus	Spec/Kan
**pRATIO4214**	*UBQ10p*	-	mScarlet-I	*F2A	mCerulean	Spec/Kan
**pRATIO4231**	*UBQ10p*	-	mNeonGFP	*F2A	mScarlet-I	Spec/Kan
