# Supplementary material for: A series of dual‐reporter vectors for ratiometric analysis of protein abundance in plants
Source: Plant Direct. 2020 Jun 21;4(6):e00231. doi: 10.1002/pld3.231 (PMC7306620; doi:10.1002/pld3.231)
Supplement: Supplementary file 3 — Table S2 [file PLD3-4-e00231-s003.pdf]

Table S2. Fluorescent proteins (FPs) used in this study.

	Optimal filter settings for tobacco			Type	Brightness	Photostability	Maturation[e]
FP	Excitation	Dichroic	Emission		E X QY[f]	$t_{1/2}$ (s)[g]	t_{50} (min)[h]
mScarlet-I[a]	560-10	573.5	595-10	m	57	225	36
Venus[b]	497-15	517.2	540-20	m	53	15	17.6
mNeonGreen[c]	505-10	522.5	540-10	m	92.8	158	10
mCerulean[d]	420-10	446.5	473-10	m	17	NA	6.6

[a]Bindels et al. 2017, [b]Nagai et al. 2002, [c]Shaner et al. 2013, [d]Rizzo et al. 2004, [e]Balleza et al. 2018, [f] E is the extinction coefficient, QY is the quantum yield, and calculated brightness is the product of E and QY (E × QY) is their product. [g] time in seconds to bleach to half of the initial intensity at an initial emission rate of 1000 photons/s. [h]Time of half-maximal fluorescence maturation (t_{50}). NA, not determined. Type: m, monomer.
