# Supplementary material for: A series of dual‐reporter vectors for ratiometric analysis of protein abundance in plants
Source: Plant Direct. 2020 Jun 21;4(6):e00231. doi: 10.1002/pld3.231 (PMC7306620; doi:10.1002/pld3.231)
Supplement: Supplementary file 4 — Table S3 [file PLD3-4-e00231-s004.pdf]

Table S3. Luciferases used in this study.

Luciferase	Origin	Optimal filter settings	Substrate
LUC2	*Photinus pyralis*	580-80	D-Luciferin
redLUC	*Luciola cruciata*	640-20	D-Luciferin
gLUC	*Gaussia princeps*	480-80	Coelenterazine
