# Supplementary material for: A series of dual‐reporter vectors for ratiometric analysis of protein abundance in plants
Source: Plant Direct. 2020 Jun 21;4(6):e00231. doi: 10.1002/pld3.231 (PMC7306620; doi:10.1002/pld3.231)
Supplement: Supplementary file 5 — Table S4 [file PLD3-4-e00231-s005.pdf]

Table S4. Primers used in this study.

Name	5’-3’ sequence
mScarlet_ApaI-F	CAT TCG CG**G GGC CCA** ATG GTG TC
mScarlet_MluI-R	CAGTGAATTCGAG**ACGCGT**CTTGTACAACT
Venus_KpnI-F	GTACAAAGTG**GGTACC**ATGGTGAGCA
Venus_SacI-R	CTT**GAGCTC**TTAGGCCGCTGCAGCAAT
KAI2_GW-F	GGGGACAAGTTTGTACAAAAAAGCAGGCTTCATGGGTGTGGTAGAAG
KAI2_GW-R	GGGGACCACTTTGTACAAGAAAGCTGGGTGTCACATAGCAATGTCATTAC
S95A_Inf-F	GGCCACGCTGTTTCTGCCATGATT
S95A_Inf-R	AGAAACAGCGTGGCCAACAAA

Nucleotide bases shown in bold denote restriction sites used for cloning
