# Supplementary material for: A series of dual‐reporter vectors for ratiometric analysis of protein abundance in plants
Source: Plant Direct. 2020 Jun 21;4(6):e00231. doi: 10.1002/pld3.231 (PMC7306620; doi:10.1002/pld3.231)
Supplement: Supplementary file 6 — Table S5 [file PLD3-4-e00231-s006.pdf]

Table S5. GenBank accession numbers for the pRATIO vectors.

Vector name	GenBank accession No.
pRATIO1112	MT024582
pRATIO1131	MT024581
pRATIO1151	MT024579
pRATIO2112	MT024574
pRATIO2131	MT024573
pRATIO2151	MT024572
pRATIO1212	MT024583
pRATIO1251	MT024580
pRATIO1267	MT024584
pRATIO2212	MT024578
pRATIO2214	MT024575
pRATIO2231	MT024577
pRATIO2251	MT024576
pRATIO3212	MT024588
pRATIO3267	MT024589
pRATIO4212	MT024587
pRATIO4214	MT024585
pRATIO4231	MT024586
